# Prognosis of Zero Coronary Artery Calcium Score in Symptomatic Patients of South Asian Descent – an Experience from a Tertiary Care Center in Pakistan

**DOI:** 10.5334/gh.1365

**Published:** 2024-10-28

**Authors:** Pirbhat Shams, Fateh Ali Tipoo Sultan, Aiman Sultan, Umair Javed

**Affiliations:** 1 Aga Khan University, Pakistan

**Keywords:** South Asian, CTCA, Zero CAC, Agatston score, Pakistan, MACE

## Abstract

**Introduction:**

The absence of CAC in asymptomatic individuals is associated with a very low incidence of cardiovascular events. Of symptomatic patients, 1–2% with zero CAC score have non-calcified coronary artery atherosclerosis, and at least one third of cardiovascular events occur in individuals with zero CAC. South Asians (SA) have proportionally higher case fatality rates for CVD, relatively younger age of presentation, and accelerated rate of atherosclerosis when compared with other ethnic groups.

**Methods:**

All consecutive patients who underwent a CTCA to evaluate angina or angina-equivalent symptoms during the study duration were enrolled retrospectively. Patients with prior myocardial infarction, history of revascularization, and congenital heart disease were excluded. MACE was defined as the total of cardiac death, non-fatal myocardial infarction, and/or non-elective revascularization.

**Results:**

A total of 534 patients were enrolled after final exclusion. The mean age was 53 years ± 11. Males constituted 68.4% of the study population. Dyslipidemia was the most common co-morbid condition identified (50%), followed by diabetes (18.4%) and hypertension (3.6%). At least 28.8% of patients with zero CAC scores had the presence of coronary artery disease (soft plaque) of any degree. Obstructive CAD (>50%) was present in 5.8% of patients. Follow-up was available for 61.4% of patients. On a mean follow-up of 96.6 months ± 49.8 (range 21–194 months), all-cause MACE was observed in 8.8% of patients. The most common MACE was angina (3.96%) and all-cause mortality (3%). The baseline characteristics and MACE did not differ in patients with and without obstructive CAD. The baseline characteristics did not differ significantly between patients with and without MACE.

**Conclusion:**

The incidence of soft plaque in this SA cohort is higher than that reported in international studies. However, in symptomatic SA, a CAC score of zero carries a good long-term prognosis, irrespective of the degree of CAD.

## Introduction

Coronary artery calcification is a well-established marker of future cardiovascular risk (1). Cardiac CT is widely used for the evaluation of chest pain due to it being a non-invasive, cost-effective, and highly sensitive technique (2). CT calcium scoring detects and quantifies coronary artery calcium (CAC) and CT coronary angiography (CTCA) allows for detailed anatomical evaluation of luminal stenosis secondary to both calcified and non-calcified atherosclerotic plaques (2).

The absence of CAC (defined as a zero CAC score on CT) in asymptomatic individuals is associated with a very low incidence of cardiovascular events over a 15-year follow-up (3). However, 1–2% of symptomatic patients with chest pain and a zero CAC score have non-calcified coronary artery atherosclerosis (4, 5). The long-term prognosis of these symptomatic patients with a zero CAC score remains unclear (6). Wang et al. (7) showed that a zero CAC is associated with good negative predictive value for ruling out obstructive CAD (more than 50% stenosis) and a good prognosis despite the presence of obstructive CAD on CTCA.

South Asians (SA) have proportionally higher case fatality rates for cardiovascular diseases (CVD), relatively younger age of presentation, and accelerated rate of atherosclerosis when compared with other ethnic groups (8). When compared with the other groups in the Mediators of Atherosclerosis in South-Asians living in America (MASALA) study, SA had greater incidence and progression in CAC score and had greater change than Chinese, Black, and Latino men, but similar to Whites (9). This brings us to the need to look at the prognosis of symptomatic patients with zero calcium scores at our center.

## Objectives

The objectives of this study were to estimate the prevalence of non-calcified coronary artery disease in patients with chest pain and a zero coronary artery calcium score, and to assess the prognostic significance of a zero coronary artery calcium score in these symptomatic patients.

## Methods

This was a retrospective study conducted at a tertiary care center of a large metropolitan city in Pakistan. All consecutive patients who underwent a CT coronary angiogram (CTCA) for evaluation of angina-like or angina-equivalent symptoms from 2009 to December 2020 were enrolled. Asymptomatic patients undergoing CTCA were excluded from the analysis. Patients with prior myocardial infarction, revascularization (percutaneous coronary intervention or coronary artery bypass graft), and congenital heart disease were excluded. Patients referred for a cardiac CT scan for non-coronary indications such as arrhythmias, aortic valve, and pericardial evaluations were also excluded.

Clinical and basic demographic data was obtained by reviewing the hospital’s electronic patient record system. After reviewing the medical records, a predesigned data entry form was filled in for each patient. Information was collected regarding age, gender, clinical features at presentation, co-morbid conditions, and findings of CT coronary angiogram. Follow-up data was collected using the hospital’s electronic patient record system and telephone communication. The endpoint, major-adverse cardiovascular events (MACE), was defined as the total of cardiac death, non-fatal myocardial infarction, and/or non-elective revascularization. Additionally, the incidence of all-cause mortality was evaluated. The prevalence of non-calcified plaque in symptomatic patients with zero Agatston score was estimated.

CT scan was performed using Canon 64 and 320-multidetector Scanner, single number of sources, and retrospective ECG triggering. CAC was calculated using the Agatston method.

## Data analysis

Data was analyzed using Statistical Package for Social Sciences (SPSS) version 23.0.0 (IBM Corp. Released 2018). Continuous variables were presented as mean ± SD or median, and categorical variables as counts with proportions. Comparison of between-group continuous variables was performed using the unpaired t-test, while categorical variables were analyzed using the Pearson χ^2^ two-tailed test. For inferential analysis, patient data from the two study periods (i.e., with MACE and without MACE), were compared for all their characteristics using the Pearson Chi2 test and Fisher’s exact. Calculations were done separately for each variable between two patient groups with a significant p-value less than or equal to 0.05. Logistic regression univariate and multivariable analysis was done, using maximum likelihood estimation to analyze the relationship between dependent and independent variables.

## Results

**Figure 1:**
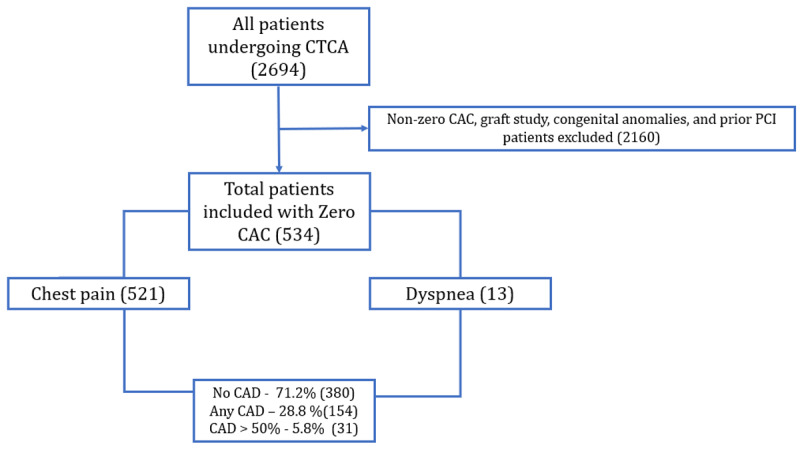
Study flow chart.

A total of 534 patients were included in the study (Figure 1). The baseline characteristics of the patients are shown in Table 1. The mean age was 53 years ± 11. Over one third of patients were males (68.4%), while 31.6% were females. Dyslipidemia was the most common co-morbid condition identified (50%), followed by diabetes (18.4%) and hypertension (3.6%). Chest pain was the most common presenting complaint (97.4%), followed by dyspnea.

**Table 1:** Baseline characteristics of symptomatic patients with zero Agatston score.


BASELINE CHARACTERISTICS	N = 534

**Mean age**	53 ± 11

**Gender, male, n (%)**	365 (68.4)

**Gender, female, n (%)**	169 (31.6)

**Hypertension, n (%)**	19 (3.6)

**Diabetes Mellitus, n (%)**	98 (18.4)

**Smoking, n (%)**	7 (1.3)

**Dyslipidemia, n (%)**	267 (50)

**Family history of CAD, n (%)**	9 (1.7)

**Symptom, chest pain, n (%)**	521 (97.4)

**Symptom, dyspnea, n (%)**	13 (2.4)

**Prior stress testing, n (%)**	189 (35.4%)

**ETT, positive**	18 (3.6)

**ETT, negative**	66 (12.3)

**MPS, positive**	5 (1)

**MPS, negative**	56 (10.9)

**Stress echo, positive**	2 (.4)

**Stress echo, negative**	13 (2.5)


**Figure 2:**
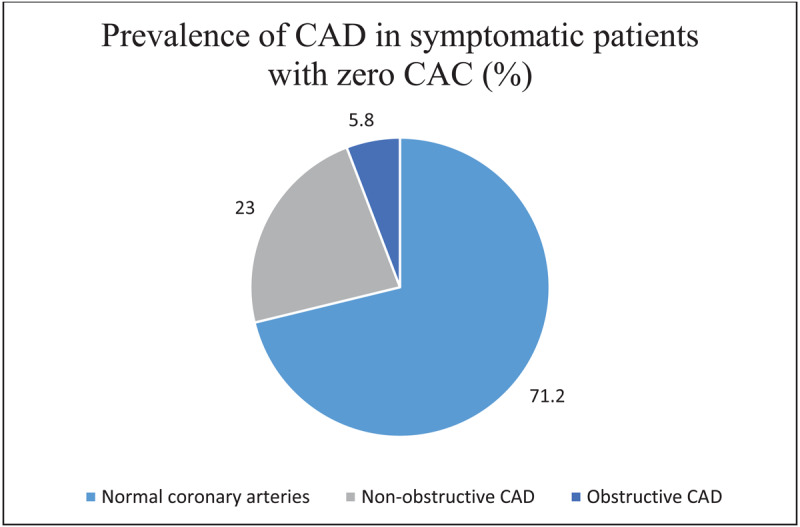
Distribution of coronary artery disease in symptomatic patients with zero Agatston score. CAC, coronary artery calcium. CAD, coronary artery disease.

At least 28.8% of patients with zero CAC scores had the presence of coronary artery disease (soft plaque) of any degree. Coronary artery disease (CAD) over 50% was present in 5.8% of patients (Figure 2). The most involved major epicardial vessel was the left anterior descending artery (LAD 22.7%), followed by the right coronary artery (RCA 11.8%). The distribution of CAD in these patients is shown in Table 2.

**Table 2:** Coronary artery disease distribution in symptomatic patients with zero Agatston score.


CTCA VARIABLES	N (%)

**CAD more than 50%, n (%)**	31 (5.8)

**Any CAD – soft plaque, n (%)**	154 (28.8)

**Left main disease, n (%)**	

**Mild**	13 (2.4)

**None**	521 (97.6)

**LAD disease, n (%)**	

**Mild**	101 (18.9)

**Moderate**	10 (1.9)

**Severe**	10 (1.9)

**None**	77.3 (77.3)

**LCx disease, n (%)**	

**Mild**	34 (6.4)

**Moderate**	2 (.4)

**Severe**	5 (.9)

**None**	493 (92.3)

**RCA disease, n (%)**	

**Mild**	52 (9.7)

**Moderate**	8 (1.5)

**Severe**	3 (.6)

**None**	471 (88.2)

**OM disease, n (%)**	

**Mild**	12 (2.2)

**Moderate**	3 (.6)

**Severe**	4 (.7)

**None**	515 (96.4)

**Diagonal disease, n (%)**	

**Mild**	20 (3.7)

**Moderate**	4 (.7)

**Severe**	5 (.9)

**None**	505 (94.5)

**Ramus, n (%)**	

**Mild**	6

**Moderate**	1

**Occluded**	1


Follow-up was available for 61.4% of patients (Table 3). The mean follow-up duration was 96.6 months ± 49.8 (range 21–194 months). All-cause Major Adverse Cardiovascular Event (MACE) was observed in 8.8% of patients. The most common MACE outcome was angina (3.96%), followed by all-cause mortality (3%). Only 2.7% of patients required revascularization on follow-up, with 1.2% having myocardial infarction and non-urgent revascularization.

**Table 3:** Outcome on follow-up.


OUTCOMES ON FOLLOW-UP	N (%)

**Follow-up available**	328 (61.4)

**Follow-up duration, mean**	96.6 months ± 49.8 (range 21–194 months),

**Mortality**	10 (3)

**Angina**	13 (3.96)

**Non-fatal MI**	4 (1.2)

**Revascularization**	9 (2.7)

**Non-elective revascularization**	4 (1.2)

**All-cause MACE (Major adverse cardiovascular events)**	29 (8.8)


**Figure 3:**
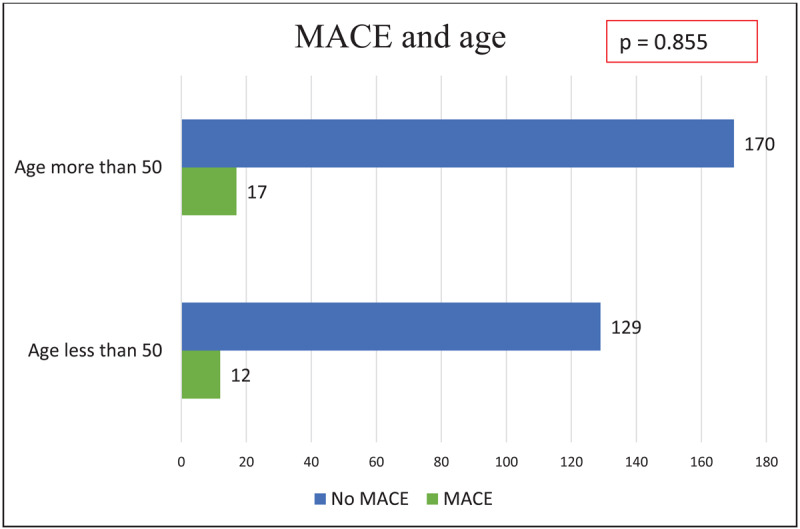
Major adverse cardiovascular events by age.

**Figure 4:**
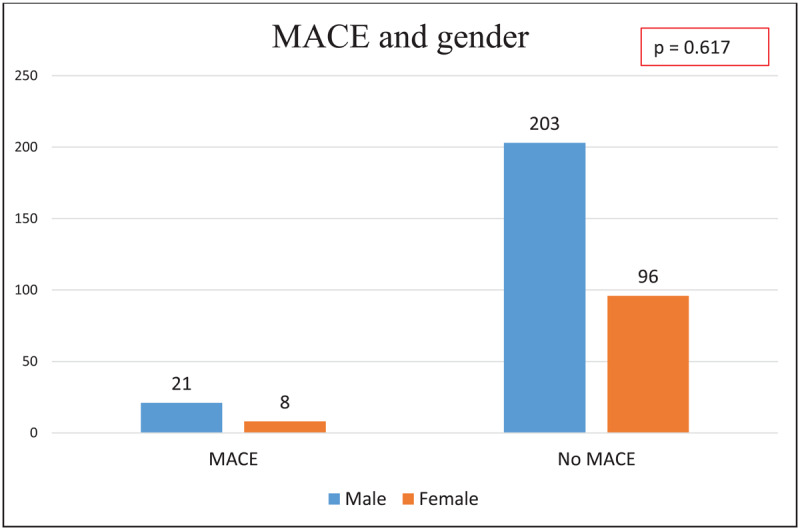
Major adverse cardiovascular events by gender.

**Figure 5:**
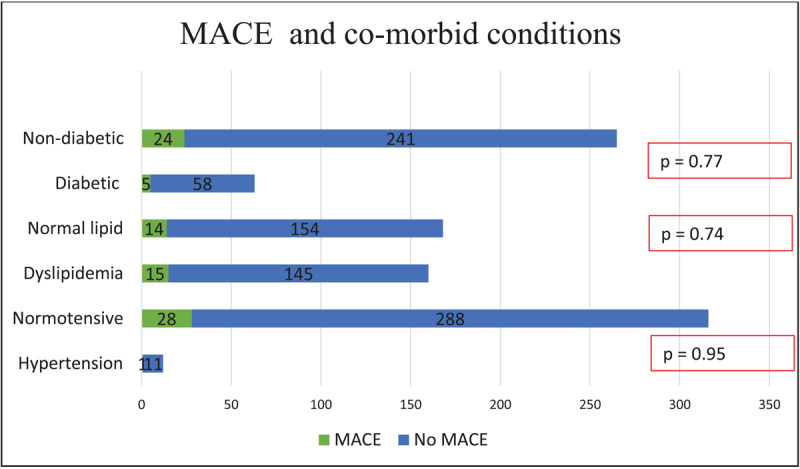
Major adverse cardiovascular events by co-morbid conditions.

On multivariate analysis, there was no significant difference in patients with MACE and without MACE, in terms of age, gender, diabetes mellitus, hypertension, symptoms, and dyslipidemia (Figures 3,4, 5). Notably, patients without MACE had a greater prevalence of obstructive CAD, which did not differ significantly from the MACE group (Table 4). Likewise, the baseline characteristics did not differ between the patients with and without obstructive CAD (Table 5).

**Table 4:** Comparison of patients with and without major adverse cardiovascular events on follow-up.


VARIABLES	MACE	NO MACE	P VALUE

**Age, more than 50 years**	17	170	0.855

**Gender, Male (n)**	21	203	0.617

**Hypertension**	1	11	0.95

**Dyslipidemia**	15	145	0.74

**Diabetes Mellitus**	5	58	0.778

**Symptoms, chest pain**	29	288	0.293

**Symptoms, dyspnea**	0	10	0.317

**CAD > 50%**	2	17	0.79


**Table 5:** Comparison of patients with and without obstructive coronary artery disease.


VARIABLES	CAD MORE THAN 50%	CAD LESS THAN 50%	P VALUE

**Gender, male**	24	341	0.263

**Gender, female**	7	162	

**Hypertension**	1	18	0.918

**Diabetes mellitus**	6	92	0.882

**Dyslipidemia**	17	250	0.579

**Family history, premature coronary artery disease**	0	9	0.453

**Chest pain**	30	491	0.768

**Dyspnea**	1	12	0.768


**Figure 6:**
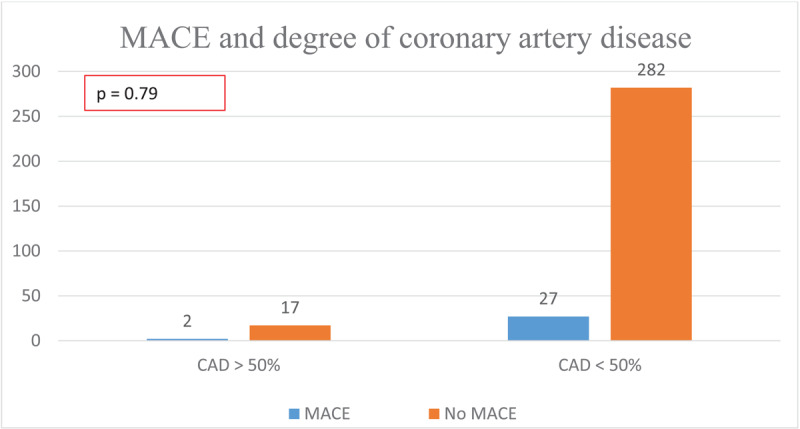
Major adverse cardiovascular events by degree of coronary artery stenosis.

On follow-up, patients with CAD < 50% had greater MACE events than those with CAD > 50%, but this did not reach a level of statistical significance (Table 6 and Figure 6). There was no significant difference in all-cause MACE (p = 0.79), mortality (0.82), angina (p = 0.765), revascularization (p = 0.45), non-fatal MI, and non-elective revascularization (p = 0.6).

**Table 6:** Outcomes of symptomatic patients with and without obstructive coronary artery disease.


OUTCOME VARIABLES	CAD > 50%	CAD < 50%	P VALUE

**All-cause MACE**	2	27	0.79

**Mortality on follow-up**	1	9	0.82

**Angina on follow-up**	1	12	0.765

**Non-fatal MI on follow-up**	0	4	0.618

**Revascularization on follow-up**	0	9	0.45

**Non-elective revascularization**	0	4	0.618


## Discussion

The presence of calcium in coronary arteries is a marker of atherosclerosis. Measurement of CAC score is a cost-effective, non-invasive, low-radiation, and quick tool for assessing sub-clinical atherosclerotic CAD (10). It carries a higher predictive value and outperforms other traditional risk factors such as carotid intima-media thickness, hs-CRP, or Framingham risk score (11).

A high CAC helps advise stringent management strategies for ASCVD risk management, whereas a CAC score of zero helps down-risk individuals and minimize statin use. In low-risk individuals, the absence of CAC is associated with a very low risk of cardiovascular events (12). Additionally, a CAC score of zero provides an incremental prognostic value in symptomatic patients without known CAD. In the results of a study conducted on 963 symptomatic patients with stable chest pain undergoing CTA at a tertiary center of East London, UK, Caucasians had a lower incidence of non-calcified plaques than South Asians, independent of age, diabetes, dyslipidemia, and other traditional cardiovascular risk factors (13).

In the secondary analysis of the Multi-ethnic Study of Atherosclerosis (MESA), at least one third of cardiovascular events occurred in individuals with zero CAC (14). On the contrary, in a large retrospective cohort of 1,145 symptomatic patients without known CAD at Massachusetts General Hospital or the Brigham and Women’s Hospital, USA, the NPV for CAC was >99% for coronary artery stenosis >50% and >99.6% for coronary artery stenosis over 70%.

In this cohort of symptomatic patients, only 1–2% of patients with zero CAC score had obstructive CAD, but this was not associated with an adverse prognosis on a two-year follow-up (5).

In a prospective cohort of 915 symptomatic patients with stable chest pain and zero CAC, at Addenbrooke’s Hospital, United Kingdom, only 1.9% of patients had obstructive CAD (>50% stenosis), and 8.4% had CAD < 50% (7). However, in our study, which was conducted at a tertiary care center of a large metropolitan city in a South Asian country, out of 534 symptomatic patients with zero CAC score, approximately 29% of patients had non-calcified plaques and 5.8% had obstructive CAD (>50%).

Results of the CONFIRM study (15), a large multi-center international registry of 10,037 symptomatic patients with zero Agatston, revealed an overall incidence of non-obstructive soft plaques (13%) and obstructive plaques (CAD > 50%) (3.5%). On a median follow-up of 2.1 years, the mortality did not differ amongst patients with zero CAC, irrespective of obstructive CAD. However, 3.9% of patients with zero CAC but obstructive CAD experienced composite endpoint (mortality, late revascularization, and myocardial infarction) when compared with patients with zero CAC and CAD < 50% (hazard ratio: 5.7; 95% confidence interval: 2.5 to 13.1; p < 0.001). A CAC score of zero did not add incremental prognostic value beyond that provided by the CAD stenosis degree on CTA.

In our study, despite a higher prevalence of soft plaques in patients with zero CAC score, the rates of all cause-MACE, mortality, angina, non-fatal myocardial infarction, and non-elective revascularization did not differ significantly between patients with obstructive and non-obstructive CAD. These results agree with the study conducted by Wang X et al. (7), in which the annualized MACE rate in patients with zero CAC was good on medium-term follow-up (1.9 per 1,000 person-year).

## Conclusion

In this symptomatic cohort of South-Asian descent with zero CAC score, the incidence of soft plaque is higher than that reported in the international studies. However, despite a high incidence of soft plaque in this symptomatic cohort, a CAC score of zero carries a good long-term prognosis, irrespective of the degree of CAD.

## Limitations

Our study had the following limitations. Patients included in the study were those who underwent testing, possibly excluding those who were symptomatic but did not receive the test. Hence, the study population may not be representative of the general population. Due to the study’s retrospective nature, researchers had limited ability to control confounding factors, such as lifestyle, diet, genetic predispositions, or socioeconomic status. Researchers had no control over the original data collection process.

The study utilized all available data for the population of interest, within the described time frame, eliminating the need to calculate a sample size or expected power of analysis in the traditional sense. All consecutive patients within the time frame, meeting the inclusion criteria were enrolled. Since this was a retrospective study, the available records predetermined the sample size. We suggest that future research could address this issue with prospective designs.

Our study found a higher burden of soft plaques in South Asians with a zero CAC score, but still reported a good prognosis. Hence, it is likely that the small sample size played a role. We suggest that our study be replicated with a larger sample size so that a more representative population is available to assess the impact of soft plaques on prognosis in South Asians.

## Data accessibility statement

Data are available on reasonable request.
